# Reevaluation of ANS Binding to Human and Bovine Serum Albumins: Key Role of Equilibrium Microdialysis in Ligand – Receptor Binding Characterization

**DOI:** 10.1371/journal.pone.0040845

**Published:** 2012-07-19

**Authors:** Irina M. Kuznetsova, Anna I. Sulatskaya, Olga I. Povarova, Konstantin K. Turoverov

**Affiliations:** 1 Laboratory of Structural Dynamics, Stability and Folding of Proteins of the Institute of Cytology, Russian Academy of Sciences, St. Petersburg, Russian Federation; 2 Department of Biophysics, of St. Petersburg State Polytechnical University, St. Petersburg, Russian Federation; University of Hyderabad, India

## Abstract

In this work we return to the problem of the determination of ligand–receptor binding stoichiometry and binding constants. In many cases the ligand is a fluorescent dye which has low fluorescence quantum yield in free state but forms highly fluorescent complex with target receptor. That is why many researchers use dye fluorescence for determination of its binding parameters with receptor, but they leave out of account that fluorescence intensity is proportional to the part of the light absorbed by the solution rather than to the concentration of bound dye. We showed how ligand–receptor binding parameters can be determined by spectrophotometry of the solutions prepared by equilibrium microdialysis. We determined the binding parameters of ANS – human serum albumin (HSA) and ANS – bovine serum albumin (BSA) interaction, absorption spectra, concentration and molar extinction coefficient, as well as fluorescence quantum yield of the bound dye. It was found that HSA and BSA have two binding modes with significantly different affinity to ANS. Correct determination of the binding parameters of ligand–receptor interaction is important for fundamental investigations and practical aspects of molecule medicine and pharmaceutics. The data obtained for albumins are important in connection with their role as drugs transporters.

## Introduction

Correct determination of ligand-receptor binding stoichiometry and binding constants is very important both for fundamental investigations and practical aspects of molecule medicine, pharmaceutics and at the development of biosensor systems of high social significance [1], [2], [3], [4], [5], [6]. The determination of binding parameters significantly enriches tried-and-true method based on fluorescence of extrinsic dyes which is widely used in molecular and cellular biology for investigation of protein’s folding, structural changes induced by different agents, interaction with each other, aggregation, amyloid fibril formation etc. In recent years it is more and more extensively used for practical diagnostics due to significant advances in molecular medicine. In all cases the knowledge of binding parameters is difficult to overestimate. The use of fluorescent dyes is based on their ability to form a highly fluorescent complex with target receptor [7], [8]. That is why it seems natural that many scientists used fluorescence for characterization dye–receptor interaction. In almost all of the studies focused on this problem, the binding constants and binding stoichiometry were evaluated on the basis of the dependence of dye fluorescence intensity on its or receptor concentration (see e.g. [1], [3], [9], [10], [11], [12], [13], [14], [15]). These works were based on the assumption that the fluorescence intensity as a function of dye concentration reaches a plateau when all of the binding sites are occupied [3], [16]. In this work, we demonstrate that the dependence of fluorescence intensity on optical density of solution is always the curve with saturation for any fluorophore (even without receptor). We show that fluorescence intensity depends not only on optical density of fluorophore but on total optical density of solution and proposed how it can be corrected on this value. And finally one must not forget that fluorescence intensity depends also on fluorophore molar extinction coefficient and its quantum yield which are not equal for free and bound dye. We for the first time determined these values for 1-anilino-8-naphthalene sulfonate (ANS) bound to proteins. In this work we showed how problems that cannot be solved by fluorescence can be solved by spectrophotometric determination of the concentration of bound dye if sample and reference solutions are prepared by equilibrium microdialysis. The knowledge of the concentration of bound dye gives an opportunity of correct determination of binding stoichiometry and binding constants. Furthermore, only the use of equilibrium microdialysis for preparation reference solution appropriate for the sample solution allows to determine absorption spectrum of bound dye and its molar extinction coefficient. Finally, fluorescence intensity of the same solutions can be used for determination quantum yield of bound dye and even fluorescence quantum yields of the dye bound to sites of different binding modes. All said above we illustrated by ANS–serum albumins interaction.

The interaction of ANS with proteins was recognized almost 60 years ago [17]. The dye has a low fluorescence quantum yield in polar environments, which is greatly enhanced on interaction with many proteins. ANS was used as a probe for biological membranes studies [18], for the analysis of conformational changes, folding–unfolding processes in proteins and molten globule test [19]. In the work [20] we make an assumption that ANS binds to the aggregates of proteins in molten globule state rather than to the hydrophobic clusters on the surface of a protein in the molten globule state. In several works the possibility of charge interaction of ANS with proteins is mentioned [20], [21], [22]. In the work [23] it was shown that ANS binds to a solvent exposed region and take part in formation of a hydrophobic regions (clusters, environment), though in its environment positively charged arginine residue was found, also. As a hydrophobic probe it has been frequently used to study the binding properties of hydrophobic species of albumins [24], [25]. Serum albumins are the most predominant proteins in blood plasma. The extensive study of these proteins is connected with their unique ability to bind and transfer a wide variety of different substances such as metal ions, metabolites, fatty acids, others biologically active species including drugs, etc. [26], [27].

## Materials and Methods

### 1. Reagents

ANS from Serva (Germany), quinine sulfate (QS) from AnaSpec (Fremont, California, USA), HSA from (Ronkonkoma, NY, USA) and BSA from Koch-Light Lab Ltd (Coinbrook, Bucks., U.K.) were used without afterpurification. HSA was dissolved in 20 mM phosphate buffer (pH 6.3). BSA was dissolved in 10 mM PBS buffer (pH 7.4) [3]. Fluorescence measurements were performed with homemade spectrofluorimeter [28] and spectrofluorimeter Cary Varian Eclipse (Mulgrave, Victoria, Australia). The solution of QS in 0.5 M H_2_SO_4_, whose fluorescence and absorption spectra are similar to that of ANS, was taken as a reference for determining the fluorescence quantum yield of ANS bound to proteins.

### 2. Fluorescence and Spectrophotometric Measurements

Fluorescence of ANS and QS was excited at 365 nm and recorded at 470 nm. The spectral slits width was 5 nm in all experiments. Change of spectral slits width did not influence on the experimental results.

For determination the fluorescence quantum yield of the dye bound to receptor the correction coefficients κ(*λ*_em_) for QS and for the ANS – BSA and ANS – HSA solutions were determined:
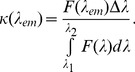
(1)Here *F*(*λ*_em_) is the fluorescence intensity at the fixed wavelength of registration and 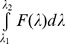 is the total fluorescence intensity. These coefficients were used for calculation total fluorescence intensity of ANS-BSA and ANS-HSA solutions (*F_ANS_*) and for QS solution (*F_QS_*). The fluorescence quantum yield of QS was taken as 0.546 (AnaSpec Catalog # 80040, 2010).

Absorption spectra were recorded by spectrophotometer Hitachi U-3900H (Tokyo, Japan).

### 3. Equilibrium Microdialysis

Equilibrium microdialysis was performed with a Harvard Apparatus/Amika (Holliston, Massachusetts, USA) device, which consists of two chambers (500 µL each) separated by membrane (MWCO 10,000) impermeable to particles larger than 10,000 Da. Equilibrium microdialysis implies allocation of two interacting agents, a ligand and receptor, in two chambers (#2 and #1, respectively) divided by a membrane permeable to the ligand and impermeable to the receptor. After that the microdialysis device are put on a rocking-bar in a thermostatted box for 48 hours. All experiments were performed at 23°C.

In the test experiments, we put a solution of ANS of concentration *C*_0_ in chamber # 2 and the solvent in chamber # 1. After 24 h of dialysis, the absorption spectra of the samples from chambers # 1 and # 2 coincide (*D*_#1_(*λ*) = *D*_#2_(*λ*) = 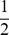 *D*_0_(*λ*)). This means that 24 h is enough time to allow the dye to equilibrate between chambers # 1 and # 2 and that ANS does not interact with membrane or chambers walls.

## Results and Discussion

The interaction of any ligand to receptor is characterized by several binding parameters: the number of binding modes, stoichiometry and binding constants of each binding mode. In the case when ligand binding sites on receptor are identical and independent of each other, the binding constant of the ligand to receptor (*K_b_*) is determined by the ratio of the ligand *–* receptor complex concentration, i.e. concentration of bound ligand (*C_b_*), to the product of the concentration of free binding sites of receptor (*nC_p_ – C_b_*) and concentration of free ligand (*C_f_*):
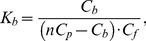
(2)where *C_p_* is the receptor concentration and *n* is the number of ligand binding sites on the receptor. Consequently, *nC_p_* is the concentration of binding sites. The value of the binding constant, *K_b_*, and the number of ligand binding sites on the receptor, *n,* can be determined on the basis of the experimental dependence of *C_b_* on *C_f_*.

The only assumption made in our work was the independence of binding sites. When processing in the frame of one mode failed to satisfy the experimental data we proposed the existence of two binding modes. The determined binding parameters of ANS-HSA and ANS-BSA interaction fit well this model. It should be noted that this model is an approximation and more binding modes can exist. The use of any more complicated model, e.g. Adair model [3], [29] which determines the sequential binding of ligands to acceptor, needs special justification.

### 1. Results that can be Obtained on the Basis of Equilibrium Microdialysis

The concentration of bound component can be determined by absorption spectrophotometry if the concentration the dye in reference solution equals to its concentration in sample solution. These solutions can be prepared by equilibrium microdialysis. Surprisingly, this approach, which is inherently designed for determining dye–receptor binding stoichiometry, has rarely been used, and only in a truncated form. The proposed approach permits to determine not only the concentration of bound and free dye but also the absorption spectra and molar extinction coefficients of dye bound to protein. We showed that fluorescence quantum yield of the dye bound to the sites of the different binding modes can be determined on the basis of the fluorescence intensity of the solutions prepared by equilibrium microdialysis.

#### 1.1. Determination of the dependence of C_b_ on C_f_

Equilibrium microdialysis implies allocation of two interacting agents, a ligand and receptor, in two chambers (#2 and #1, respectively). In our case the solution of protein with an initial concentration *C_p_*, was placed in chamber #1, and the solution of ANS in the same buffer with an initial concentration *C_0_* was placed in chamber #2. After equilibration, the concentrations of free dye in chambers #1 and #2 become equal (*C_f_*), while the total ANS concentration in chamber #1 is greater than that in chamber #2 by the concentration of the bound dye (*C_b_*), thus:
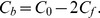
(3)


The initial concentration of ANS in chamber #2 (*C_0_*) and the dye concentration in this chamber after equilibration (*C_f_*), which equals the concentration of free dye in chamber #1, can be determined by absorption spectrophotometry. The concentration of the dye bound to protein can be determined on the basis of eq. 3.

#### 1.2. Determination of binding parameters: the number of binding modes, stoichiometry and binding constants

**Figure 1:**
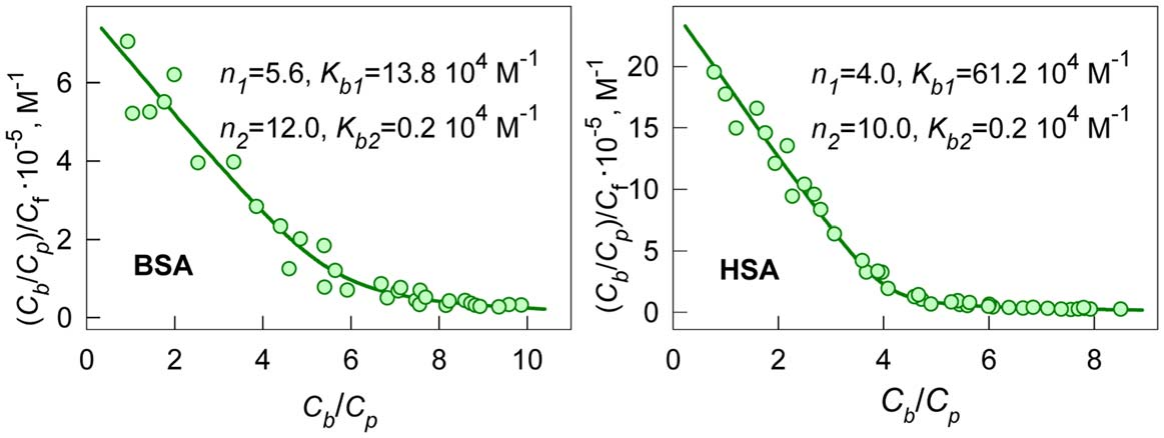
Scatchard plots for ANS interaction with BSA and HSA. Experimental data (circles) and best fit curve with binding constants (*K_bi_*) and number of binding sites (*n_i_*) given on the panels.

Equilibrium microdialysis was done for a large number of different initial ANS concentrations (*C_0_*) with subsequent spectrophotometric determination of the free (*C_f_*) and on the basis of eq. 3 bound dye concentration (*C_b_*). Traditionally these data are presented as Scatchard plot:
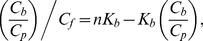
(4)which allows graphical determination of binding parameters. The nonlinear character of Scatchard plots for ANS bound to HSA and BSA (Figure 1) indicate on the existence of more that one binding mode. If the binding sites are independent of each other, 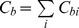 and equation similar to eq. 2 can be written for each mode:


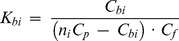
(5)

Then the dependences of *C_b_* on *C_f_* will be as follows:
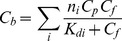
(6)


This model gives adequate description of the experimental data for ANS binding to HSA and BSA at *i* = 2. The values of *K_bi_* and *n_i_* were found as the best fit of eq. 6 using GraphPad Prism 5. The values of *K_bi_* and *n_i_* are given in Figure 1 and in Table 1. The ANS binding constants of the sites with higher affinity is about 70 and 300 times greater than that of the sites with lower affinity for BSA and HSA, respectively. The binding constant of the mode with largest affinity for HSA is more than four times greater than that for BSA. The number of binding sites with high affinity are 4 and 6 for HSA and BSA, respectively.

**Table 1:** Characteristics of ANS – BSA and ANS – HSA interaction.

protein	*mode*	*λ_max_, nm*	*ε_i, max_* 10^−3^, M^−1^cm^−1^	*ε_i, 365_* 10^−3^, M^−1^cm^−1^	*K_bi_* 10^−4^, M^−1^	*n*_i_	*q_i_*
BSA	1	374.5±0.5	5.7±0.2	5.4±0.2	13.8±0.5	5.6±0.8	0.45±0.01
	2	376.0±0.5	5.2±0.3	4.8±0.4	0.2±0.2	12.0±1.0	0.08±0.03
HSA	1	373.5±0.5	5.5±0.1	5.3±0.1	61.2±0.7	4.0±0.1	0.42±0.01
	2	374.0±0.5	5.6±0.2	5.3±0.2	0.2±0.1	10.0±0.2	0.07±0.01

#### 1.3. Absorption spectrum of ANS bound to HSA and BSA

**Figure 2:**
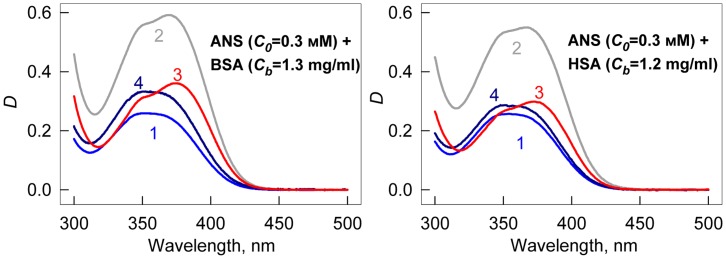
Absorption spectra of ANS bound to BSA and HSA. Curves 1 and 2 represent absorption spectra of ANS in chamber #2 (free ANS in concentration *C_f_*) and in chamber #1 (superposition of absorption spectra of free ANS in concentration *C_f_*, ANS bound to proteins in concentration *C_b_*) after reaching equilibrium. Curve 3 is the absorption spectra of ANS bound to proteins. Curve 4 is the absorption spectra of free dye in a concentration equal to that of bound dye.

At equilibrium, the absorption spectrum of the solution in chamber #2 represents the absorption spectrum of free ANS at a concentration of *C_f_* (*D_f_* (λ)), and the absorption spectrum of the solution in chamber #1 represents the superposition of the absorption spectra of free ANS (concentration *C_f_* ) and ANS bound to protein at a concentration of *C_b_* (*D_b_*(λ)). Thus, in chambers #1 and #2, we have a sample and optimal reference solutions for determination of the absorption spectrum of ANS bound to proteins. The analysis of these spectra (Figure 2) shows that the absorption spectrum of ANS bound to HSA and BSA are significantly red-shifted in comparison to that of free ANS. It is evident that the absorptioin spectra of ANS bound to HSA and BSA are complex. Apparently, it is determined by overlapping of two spectral bands.

#### 1.4. Molar extinction coefficient of ANS bound to HSA and BSA

In the case of one binding mode, the measured absorption spectrum can easily be presented in the units of the molar extinction coefficient (Lambert-Beer law). If there are two binding modes, then the concentrations of dye bound to the sites of each mode can be calculated on the basis of the *K_d1_*, *K_d2_*, *n*_1_ and *n*_2_ values:
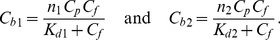
(7)


**Figure 3:**
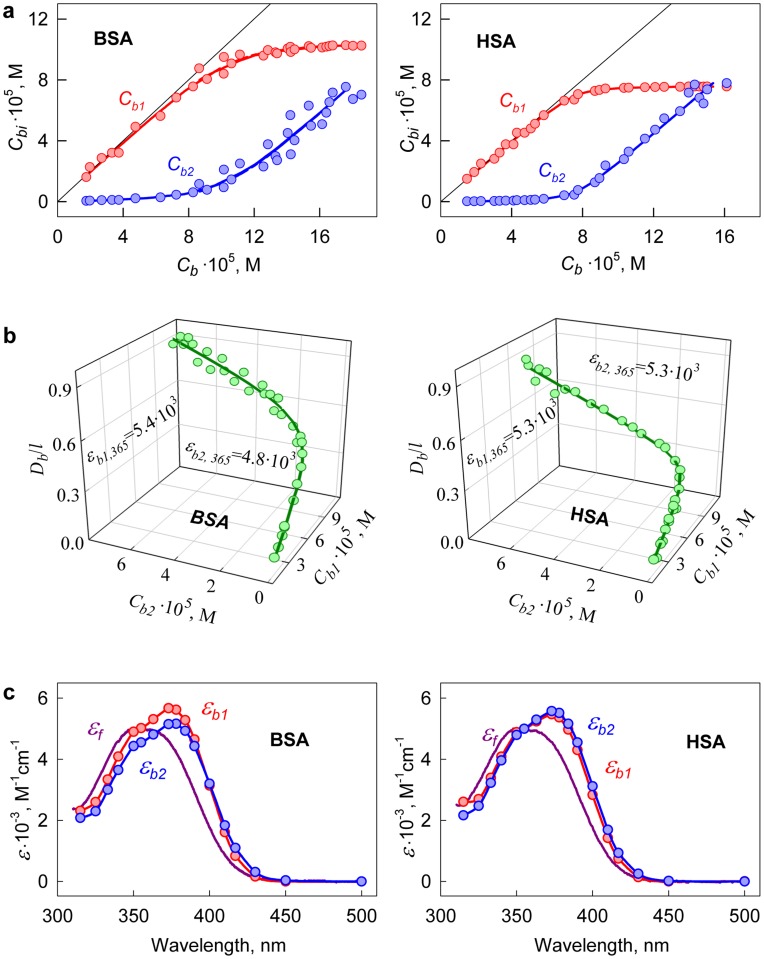
Determination of the molar extinction coefficient of ANS bound to BSA and HSA. (*a*) Concentration of ANS bound to proteins (*C_b_*) as a superposition of the concentrations of the dye bound to the sites of mode 1 (*C_b1_*) and mode 2 (*C_b2_*). (*b*) The dependence 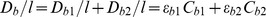 on *C_b1_*, *C_b2_*. In the panel experimental data, best-fit curve and the values of molar extinction coefficients *ε_b1_* and *ε_b2_* obtained by multiple nonlinear regression are presented. (*c*) Absorption spectra of ANS bound to the sites of mode 1 and mode 2 in the units of the molar extinction coefficient.

Figure 3a shows the decomposition of 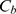 into two components, 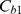 and 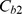, for ANS-HSA and ANS-BSA interaction. Taking into account that.
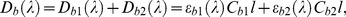
(8)the values of 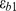
(λ) and 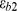
(λ) can be determined using the known values of 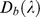, 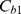 and 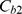 by multiple linear regression (e.g., using Graph Pad Prism 5). Figure 3b shows the relation between *D_b_* (at λ = λ_ex_ = 365 nm) and *C_b1_* and *C_b2_* values that result in values of 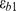 and 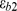 for ANS bound to HSA and BSA. Similarly, the values of 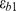 and 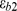 can be determined at the other wavelengths. Figure 3c shows the absorption spectra of the dye bound to the sites of each of the two modes of protein in units of the molar extinction coefficient. These data show that ANS binding to proteins is accompanied not only by the change of the position of its absorption spectra but by the change of its molar extinction coefficient, also.

### 2. Can Equilibrium Microdialysis be Replaced by Fluorescence?

**Figure 4:**
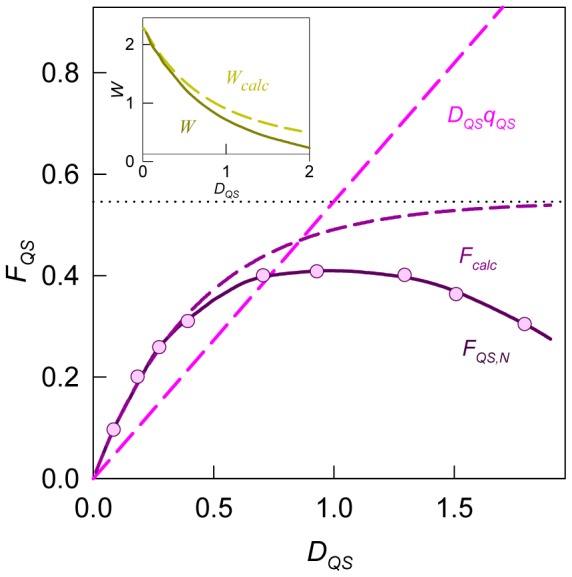
The dependences of fluorescence intensity of quinine sulfate (QS) on its optical density. 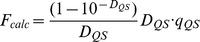
 are calculated values and 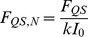 are experimentally recorded and normalized values of fluorescence intensity of QS. Dots are the limiting value of 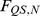 at 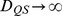. The dependences of calculated values 
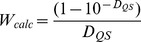
 and experimentally determined values 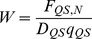 on total optical density are given in the Insert. Strait line is the dependence of 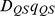 on 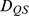 (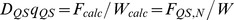).

Frequently, the external dyes are used as a common tool to study the receptor’s structure. For example, fluorescent dye ANS is usually used for the study of the process of protein’s folding, detection partially folded proteins state known as molten globule and examining their properties [19], [20], [30], [31], and fluorescent dye thioflavin T is used for detection and study of amyloid fibrils [32], [33], [34], [35], [36]. These dyes do not fluoresce in the free state, more exactly have very low fluorescence quantum yield in free state, and form highly fluorescent complexes while binding to receptor. That is why it seems natural to use fluorescence of bound dyes for determination of their binding parameters. Many researchers being not specialists in fluorescence assumed that fluorescence intensity as a function of dye concentration reaches a plateau when all of the binding sites are occupied. In reality, the dependence of fluorescence intensity on dye concentration is the curve with saturation for any (even unbound) fluorophore and it is linear only in the limit when dye concentration tend to zero (Figure 4). Furthermore, the researchers did not take into account that fluorescence intensity depends not only on bound fluorophore concentration but also on its fluorescence quantum yield and molar extinction coefficient which usually differ from that of free dye.

#### 2.1. Dependence of the fluorescence intensity on the optical density of the fluorescence substance and on the total optical density of the solution

Any ligand–receptor solution contains ligand molecules both free and bound to receptor. So the solution of ANS in the presence of serum albumins is a two-component system in which one component (free dye unbound to protein) absorbs the excitation light (its optical density is *D_f_*) but does not fluoresce, while the other component (dye bound to protein) absorbs the excitation light (its optical density is *D_b_*) and fluoresces with a quantum yield *q_b_*. In this case the fluorescence intensity of solution *F_ANS_*, excited by light with fluorescence intensity *I_0_* can be presented as follows:
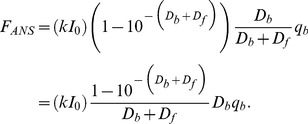
(9)


Here *k* is the proportionality coefficient. If protein has several binding sites of ANS which are independent of each other but can be divided into several groups (binding modes) which differ by the value of binding constants (*K*_b*i*_), binding stoichiometry (*n_i_*) or by the properties of bound dye (absorption spectra *D*_bi_(*λ*), molar extinction coefficients (*ε_i_*), fluorescent quantum yields (*q*_b*i*_)) then eq. 9 will be as follows:
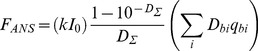
(10)


Here 
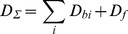
 is total optical density of solution. The fluorescence intensity of a single-component solution can be presented in the same manner. In particular, for the fluorescent dye quinine sulfate (QS), which was used in this work as a standard substance with known quantum yield, the fluorescence intensity is:
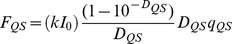
(11)


Thus in the equations 9–11 for fluorescence intensity three factors can be marked out:

- the factor *kI_0_* depends only on the device used in the experiment. It is determined by the intensity of the excitation light, the wavelength of excitation and registration of fluorescence, the spectral slits width of the monochromators in the excitation and registration pathways, sensitivity of photodetector and signal amplification.
- the factor 
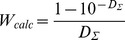
 is determined by total optical density (*D_Σ_*) of solution (at λ = λ_ex_) and does not depend on the contribution in absorption of fluorescent and non-fluorescent components of solution. The calculated dependence of this factor on the optical density of QS solution (*D_QS_ = D_Σ_*) is given in Figure 4, Insert.

The experimental dependence of fluorescence intensity on the optical density of the fluorescent substance can differ from the calculated one (Figure 4). The fact that the recorded fluorescence intensity, after reaching some value of optical density, begins to decrease with an increase in the content of the fluorescent substance is a general property of such dependences and does not indicate the existence of self-quenching or dye aggregation, as it has been frequently suggested (see e.g. [37], [38], [39]). In reality, this effect is determined by an increased absorption of excitation light by the solution layers adjacent to the front wall of the spectrofluorimeter cell on the increase in the total optical density of the solution. The detection system of the spectrofluorimeter “sees” only the central part of the cell, which is reached by a respectively smaller amount of excitation light. Because of this effect, the recorded fluorescence intensity begins to diminish after the optical density reaches a certain value. The effects discussed above depend on the particular instrument used in the experiment and must be taken into account. This can be performed by replacing *W_calc_* with an experimentally determined value *W*. The measurement of QS fluorescence intensity at different optical densities gives an opportunity to determine the dependence of *W* on total optical density (Figure 4, Insert) and to choose the normalization coefficient *kI*_0_ in order to correct the dependence of fluorescence intensity on the total optical density of solution and to normalize it in the units of the product of optical density and fluorescence quantum yield.

In fact the value of *kI*_0_ must be chosen so that *W* → *W*_calc_ at *D*_QS_ → 0 and normalized and corrected fluorescence intensity of QS (*F_QS,_*_0_) would equal to:
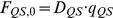
(12)

- the third factor is the product of optical density and quantum yield of the fluorescent component in the case of one binding mode or the sum of the products in the case of several binding modes. Only this factor is an informative element which bears information about the properties of fluorescent component (or fluorescent components) in solution. In this case eqs 9 and 10 for normalized and corrected fluorescence intensities (*F_ANS,_*_0_) will be as follows:

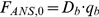
(13)or
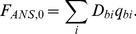
(14)


**Figure 5:**
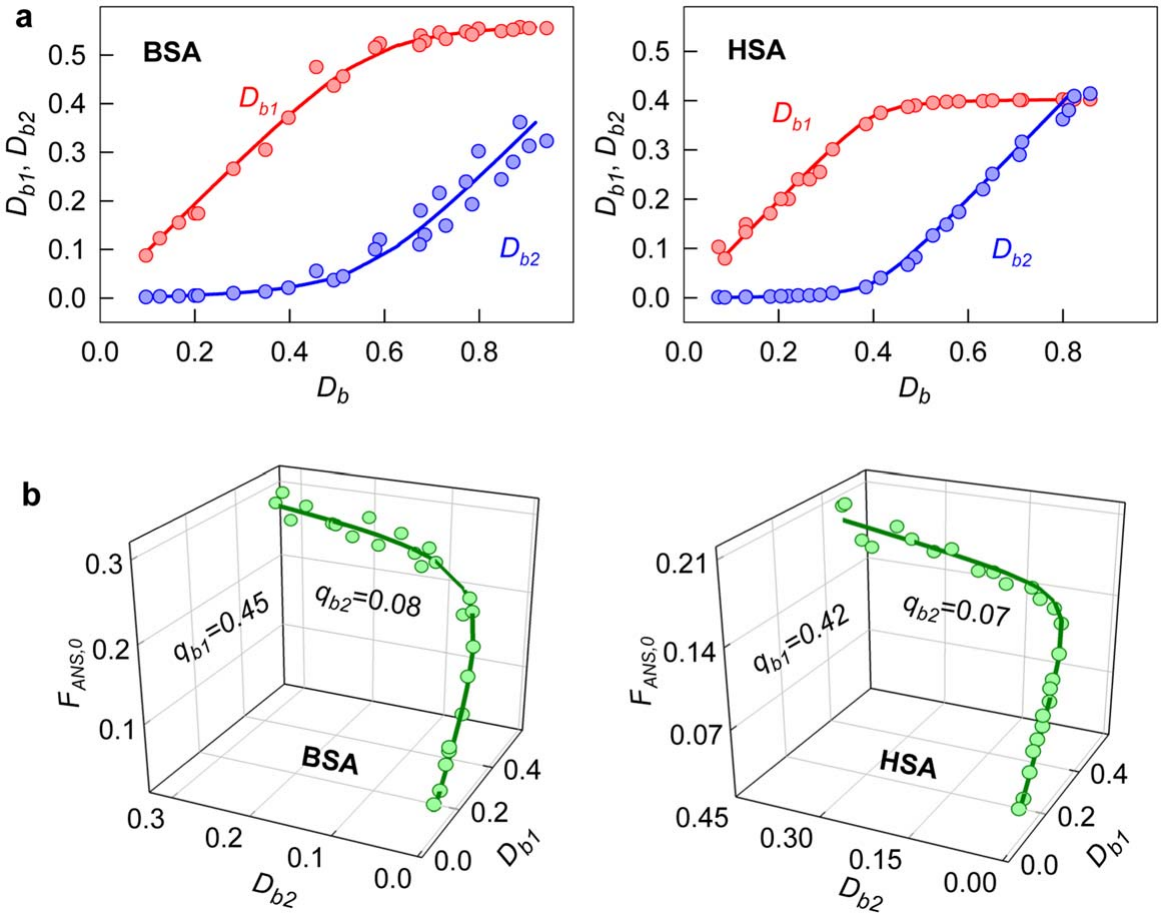
Determination of the fluorescence quantum yield of ANS bound to BSA and HSA. (*a*) Optical density of ANS bound to proteins as a superposition of optical densities of the dye bound to the sites of mode 1 (*D_b1_*) and mode 2 (*D_b2_*). (*b*) 3D dependence of 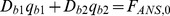 on *D_b1_* and *D_b2_*. Experimental data, best-fit curve and the values of *q_b1_* and *q_b2_*, determined by multiple non-linear regression are presented.

#### 2.2. Determination of the fluorescence quantum yield of the dye bound to proteins

If all binding sites of ligand to receptor are identical and independent of each other then fluorescence quantum yield is determined as a slope of the dependence 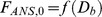 (see eq. 13). In the case of two binding modes, a relation similar to eq. (14) is valid. To determine the values of *q_b1_* and *q_b2_*, it is necessary to know the set of three related values 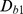, 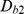 and 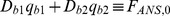, that correspond to one microdialysis experiment. The dependences of the values of 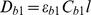
and 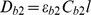 on *D_b_* can be determined on the basis of the values of the molar extinction coefficients and concentrations of ANS molecules bound to the sites of two binding modes for the samples obtained after microdialysis (Figure 5a). The dependence of the values 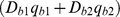 on *D_b1_* and *D_b2_* can be determined from eq. 14. On the basis of these data, the fluorescence quantum yields of the dye bound to the sites of each binding mode can be determined using multiple linear regression (Figure 5b, Table 1).

### Conclusion

In this work two significantly different binding modes were determined for ANS binding to serum albumins. The ANS binding constant of the sites with higher affinity is about 70 and 300 times greater than that with lower affinity for BSA and HSA, respectively. Fluorescence quantum yields of ANS bound with higher affinity are about 0.45 and 0.42 and that with lower affinity are about 0.08 and 0.07 for BSA and HSA. It could be thought that interaction with low affinity has nonspecific character [40] but even lower quantum yield is 20 times greater than that of free ANS in buffer solution (0.0032 [41]). This can be interpreted as the existence of several hydrophobic pockets on the surface of serum albumins which bind ANS with high affinity and numerous binding sites on the surface of proteins, perhaps, formed by hydrophobic clusters and/or charge groups which bind ANS with low affinity. The possibility of both types of ANS interaction with proteins can be found in literature [3], [4], [17], [23], [42], [43], [44], [45]. Since the first work of Weber, ANS was assigned to hydrophobic probes [17]. At the same time ANS molecule being negatively charged strongly binds to cationc groups of polyamino acids. [22] The existence of negative or positive charge groups on the surface of protein or in its hydrophobic pockets can prevent or promote its binding to protein [30], [44], [46]. ANS ability to bind hydrophobic clusters on the surface of the protein in the molten globule state make it one of the main probes for the appearance of such state [19], [45]. According to our views, ANS binds to the hydrophobic pockets in aggregates formed by proteins in molten globule state rather than with hydrophobic clusters on the surface of protein in molten globule state [20]. ANS can intercalate in the hydrophobic pockets of multidomain and/or multimeric proteins [31]. Aggregation of such proteins in the molten globule state results in the formation of new hydrophobic pockets between protein molecules that leads to the increase in number of bound ANS molecules [20], [31].

Though, there are numerous works on the investigation of ANS binding to serum albumins, yet there are no consensus about the number of binding modes, binding sites and values of binding constants [3], [12], [13], [24], [25], [42], [44], [47], [48], [49]. This is caused by significant difficulties of the binding parameters determination. Togashi and Ryder pointed that the broad range of the values of the reported binding parameters is caused by the use of different assumption and models of dye – protein interaction, the absence of the method for estimating the free ligand concentration and overlooking the true dependence of fluorescence intensity on total optical density of solution (overlooking of the inner filter effect) [3].

In our work we for the first time proposed correct estimation of the influence of the total optical density of fluorescent dye solution (free and bound dye) on its fluorescence. We showed that total fluorescence intensity is determined by the factor that is a function of total optical density of the solution only and does not depend on the relative contributions of the optical densities of the fluorescent and non-fluorescent components, and by the other factor that is a product of the optical density and the quantum yield of the fluorescent component. But even corrected fluorescence intensity is determined not only by the concentration of fluorescence dye, but also by its molar extinction coefficient and fluorescence quantum yield of bound dye that are not known and usually differ from that of free dye. For correct determination of the concentration of bound and free dye we proposed to use equilibrium microdialysis. The use of equilibrium microdialysis gave us the opportunity to determine not only the values of binding stoichiometry and binding constants but also for the first time to register absorption spectrum of bound dye and to determine molar extinction coefficient and fluorescence quantum yield of ANS bound to sites of different binding mode.
